# Development and psychometric validation of the Chinese version of Skindex-29 and Skindex-16

**DOI:** 10.1186/s12955-014-0190-4

**Published:** 2014-12-24

**Authors:** Zehui He, Chuanjian Lu, Mary-Margaret Chren, Zhongzhao Zhang, Yan Li, Xiaojia Ni, Henry A Buchtel V, Paul F Ryan, Guo-Zheng Li

**Affiliations:** Department of Clinical Epidemiology, Guangdong Provincial Hospital of Chinese Medicine, the Second Affiliated Hospital of Guangzhou University of Chinese Medicine, Guangzhou, China; Department of Dermatology, Guangdong Provincial Hospital of Chinese Medicine, the Second Affiliated Hospital of Guangzhou University of Chinese Medicine, Guangzhou, No.111 Da De Road, Guangdong, 510120 China; Department of Dermatology, University of California, San Francisco, USA; Department of Sleep and Mental Disorder, Guangdong Provincial Hospital of Chinese Medicine, the Second Affiliated Hospital of Guangzhou University of Chinese Medicine, Guangzhou, China; Department of Evidence-based Medicine and Clinical Research of Chinese Medicine, Guangdong Provincial Hospital of Chinese Medicine, the Second Affiliated Hospital of Guangzhou University of Chinese Medicine, Guangzhou, China; Michigan Association of Acupuncture and Oriental Medicine, The Lotus Center of Ann Arbor, Ann Arbor, USA; Department of Neurology, Lutheran Medical Center, New York, USA

**Keywords:** Health-related quality of life, Skindex, Chinese, Validation

## Abstract

**Background:**

Dermatological disease significantly affects patient’s health-related quality of life (HrQoL). Skindex is one of the most frequently used dermatology-specific HrQoL measures. Currently no Chinese version of Skindex is available. The aim of this study was to translate and culturally adapt Skindex-29 and Skindex-16 into Chinese, and to evaluate their reliability and validity.

**Methods:**

Translation and cultural adaption were performed following guidelines for cross-cultural adaption of health-related quality of life measures. Subsequently, a cross-sectional study was conducted in which patients with dermatological disease (n = 225) were enrolled. The Chinese version of Skindex-29 and Skindex-16 and Dermatology Life Quality Index (DLQI) were completed. Reliability was evaluated with internal consistency using Cronbach’s alpha. Validity was evaluated using known-groups validity, convergent validity and factor structure validity.

**Results:**

There were both seven items of Skindex-29 and Skindex-16 requiring a second forward- and backward- translation to achieve the final satisfactory Chinese version. The internal consistency reliability was high (range of Cronbach’s alpha for the scales of Skindex-29 0.85-0.97, Skindex-16 0.86-0.96). Known-group validity was demonstrated by higher scores from patients with inflammatory dermatosis than from patients with isolated skin lesions (P < 0.05). Evidence of factor structure validity of the Skindex-29 and Skindex-16 was demonstrated by both exploratory factor analysis that accounted for 68.66% and 77.78% of the total variance, respectively, and confirmatory factor analysis with acceptable fitness into the expected three-factor structure.

**Conclusion:**

This study has developed semantically equivalent translations of Skindex-29 and Skindex-16 into Chinese. The evaluation of the instruments’ psychometric properties shows they have substantial evidence of reliability and validity for use as HrQoL instruments in Chinese patients with dermatological disease.

## Background

Health-related quality of life (HrQoL) in dermatological patients has received growing recognition as measuring the burden of skin disease on patients in the fields of clinical practice, clinical trial, and healthcare management. In China, however, less attention has been paid to HrQoL of dermatological patients, and one of the main reasons is a lack of suitable instruments in Chinese that have been developed or adapted according to established scientific attributes and criteria [1].

Skindex is one of the best dermatological instruments to measure dermatology-specific HrQoL [2]. Skindex was comprised of 61 items initially and was then modified to two brief versions known as Skindex-29 and Skindex-16 [3-6]. The two versions have been extensively studied and refined in different languages and population samples. Skindex-29 was recommended as one of the instruments of choice in dermatology [7], and Skindex-16 was recognized as an accurate and sensitive measurement of bother of patients’ experience and used in many studies of dermatological diseases [8,9]. However, there is no Chinese version of Skindex available.

In this study, we translated into Chinese and cross-culturally adapted Skindex-29 and Skindex-16. Furthermore, assessment of psychometric properties was carried out.

## Methods

The study was carried out in two phases: translation, and evaluation of psychometric properties. The study was approved by the ethics committee of the Guangdong Provincial Hospital of Chinese Medicine.

### Translation

The translation methodology conformed to the guidelines for cross-cultural adaption of health-related quality of life measures [10]. There were 5 steps.

1. Forward-translation: Two bilingual translators whose native language was Chinese independently translated Skindex-29 and Skindex-16 into Chinese. Then the two translators discussed their translations and agreed on a reconciliation version defined as the first Chinese version.
2. Backward-translation: Two bilingual, native speakers of English who had no access to the original version of Skindex-29 and Skindex-16 independently translated the first Chinese version into English. Then a reconciliation version was agreed by the two backward-translation persons.
3. Review by original author: The first backward-translation was reviewed by the original author of Skindex-29 and Skindex-16 (MMC). The semantic equivalent of each backward-translation item was classified according to the following scale: (a) Satisfactory agreement; (b) Almost satisfactory agreement but one or two words uncertain; (c) Doubtful translation. For items classified as (b) or (c), the forward- and backward-translations were refined. The original author reviewed them again. When a satisfactory agreement with the backward-translations was reached, the second Chinese version was obtained.
4. Pre-testing: The second Chinese version was tested on a pilot group of patients with skin disease. The patients were all native speakers of Chinese. Each item was performed face to face interviews to determine whether it was acceptable and comprehensible.
5. Production of final version: The final Chinese version was produced after refining problematic items encountered in pre-testing and proof-reading.

### Evaluation of psychometric properties

#### Sample

A cross-sectional, non-interventional study of dermatological patients was conducted at the department of dermatology of Guangdong Provincial Hospital of Chinese Medicine from January to May 2013. Outpatients were recruited according to the following inclusion criteria: (1) age 16 years or more; (2) confirmed diagnosis of dermatological disease; (3) willingness to provide consent to participate. Subjects were administrated a set of questionnaires and given enough time to self-complete the questionnaires. Respondents who left greater than 25% of the items of any instrument unanswered were excluded from the evaluation phase. Subjects were categorized into those with isolated skin lesions such as nevi, warts and those with inflammatory dermatosis, such as psoriasis, acne.

#### Instruments

The measurement instruments included sociodemographic items, the validated Chinese version of Dermatology Life Quality Index (DLQI) and the final Chinese versions of Skindex-29 and Skindex-16.

The sociodemographic items included age, gender, marital status, education level, smoking, drinking alcohol, co-existing chronic disease, and duration of skin disease.

The DLQI is a 10-item brief questionnaire to assess the quality of life impact of skin disease [11]. Its Chinese version has been evaluated to have good psychometric properties [12]. It is designed as 6 aspects of quality of life: symptoms and feelings, daily activities, leisure, work and school, personal relationships and bother with treatment, while its unidimensionality has been proved for several language versions and a total score of DLQI is generally recommended to be used [13]. Items are answered on a 4-point ordinal scale ranging from 0 to 3, and item scores are summed to yield a total score (0–30).

Skindex-29 contains 30 items, while item 18 about side-effects of treatment is not scored. It covers 3 scales: symptoms, emotions and functioning. Each item is rated on a 5-point Likert scale (never, rarely, sometimes, often, all the time). All responses are transformed to a linear scale of 100 ranging from 0 to 100, and the scale score is regarded as the mean of a patient’s responses to the items in a given scale. Skindex-16 contains 16 items with each rated on a 7-point Likert scale. Scores for the emotions, symptoms and functioning scales are also expressed in a linear scale from 0 to 100, and the scoring method is the same with those of Skindex-29.

For all the three instruments used, higher scale scores reflect greater impairment.

#### Statistical analysis

For each scale of the Skindex-29 and Skindex-16, the floor and ceiling effects were assessed. If more than 20% of the participants reported lowest or highest possible score, the floor or ceiling effects exist [14].

For reliability analysis, Cronbach’s alpha coefficient was determined to assess internal consistency reliability.

Based on the hypotheses that patients with inflammatory dermatosis would have higher scale scores than patients with isolated skin lesions, known-groups validity was assessed by comparison between the two groups using Wilcoxon rank sum test. For Skindex-29, known-groups validity was also assessed by comparison among different frequency groups of bother from side-effects of treatment (measured by item 18), and the Kruskal-Wallis H test was used.

Convergent validity assessed the degree to which the Skindex-29 and Skindex-16 were similar to (converged on) other measures that they should theoretically be related to. Spearman’s rank correlation coefficient was used to assess correlations between the Skindex-29, Skindex-16 and the DLQI.

Factor structure validity was assessed by extracting factors using exploratory factor analysis (EFA) with principal components method. As substantial correlation between factors was expected, oblique rotation was performed. Factors were identified based on eigenvalues greater than 1. A criterion of highest factor loading, above 0.4 and at least 0.1 stronger than the next was used to identify items that were salient in defining a given factor. Each factor was labeled by the heavily loaded items according to the above criterion. Confirmatory factor analysis (CFA) was also used to evaluate goodness of fit of the supposed three-domain structure of the instruments. The goodness of fit indices valued between 0 and 1 included goodness of fit index (GFI), adjusted goodness of fit index (AGFI), non-normed fit index (NNFI) and comparative fit index (CFI), standardized root mean square residual (SRMR), and root mean square error of approximation (RMSEA). The first four indices were expected to be larger than 0.90, the SRMR was expected to be less than 0.08, and the RMSEA was expected to be less than 0.10 [15,16].

All the analysis was performed using SPSS 17.0 (SPSS Inc., Chicago, IL, USA) and LISREL 8.8 (Scientific Software International, Chicago, IL, USA).

## Results

### Translation

The two forward-translators developed and agreed upon the first Chinese versions of Skindex-29 and Skindex-16; the two backward-translators translated and agreed on the first versions into English, which were sent to and reviewed by the original author (MMC).

There were 23 items of Skindex-29 and 9 items of Skindex-16 classified as (a). The items that were classified as (b) or (c) were presented in Table 1 respectively for Skindex-29 and Skindex-16. The translation of some feelings and emotions such as “being frustrated”, ”being ashamed”, “being annoyed” and “showing affection” were particularly problematic.

**Table 1:** **Original and the first backward-translations of seven items in Skindex-29 and Skindex-16 with b or c classification of semantic equivalence**

	**Original**	**First-backward translation**	**Classification**
Skindex-29	11. My skin condition affects how close I can be with those I love	My skin condition affects my intimate partner/spouse	c
	12. I am ashamed of my skin condition	Because of my skin condition I feel that I lose face/don’t get respect	b
	16. Water bothers my skin condition (bathing, washing hands)	Using water affects my skin condition (i.e. bathing, washing hands)	b
	17. My skin condition makes showing affection difficult	My skin condition makes it difficult to express my emotions/feelings	c
	19. My skin is irritated	My skin is prickly/itchy	b
	23. I am frustrated by my skin condition	My skin condition makes me feel dejected	c
	28. I am annoyed by my skin condition	My skin condition makes me frustrated	b
Skindex-16	4. Your skin condition being irritated	Your skin is prickly/itchy	b
	5. The persistence/reoccurrence of your skin condition	Your skin condition continuously or repeatedly erupts	b
	6. Worry about your skin condition (For example: that it will spread, get worse, scar, be unpredictable, etc.)	You feel worried about your skin condition (i.e. the disease will spread, worsen, leave scars, be uncontrollable)	b
	8. Frustration about your skin condition	You feel dejected about your skin condition	c
	10. Being annoyed about your skin condition	You feel frustrated about your skin condition	c
	14. Your skin condition making it hard to show affection	Your skin condition makes it difficult to express your emotions/feelings	c
	15. The effects of your skin condition on your daily activities	Your skin condition affects your daily life	b

A second forward- and backward-translation procedure was performed for items with classification (b) and (c). The original author reviewed the second backward-translation of items with classification (c) and the results were presented in Table 2. For the item 23 of Skindex-29 and item 8 of Skindex-16 classified as (b), there is no Chinese word that means precisely the same with “being frustrated”. Finally, a phase was used to express the concept to achieve satisfactory agreement with the original instruments. Then the second Chinese version of the questionnaires was developed, and it was tested on a panel of 10 patients with dermatological disease aged from 20 years to 68 years old. There were no problems with comprehension of all the items. The final version was produced after a proof-reading.

**Table 2:** **The second backward-translations of three items with c classification of the first backward-translations and the new semantic equivalence classification**

	**Item**	**Second-backward translation**	**Classification**
Skindex-29	11	My skin condition affects intimate contact with loved ones.	a
	17	My skin condition makes it difficult for me to express love and affection.	a
	23	My skin condition makes me feel hopeless.	b
Skindex-16	8	Your skin condition makes you feel hopeless.	b
	10	Your skin condition makes you irritated.	a
	14	Your skin condition makes it difficult for you to express love and affection.	a

### Psychometric properties

#### Sample characteristics

A total of 225 patients with dermatological disease were enrolled. The mean age of participants was 32.5 years (standard deviation = 12.2) and 47% of participants were men. Forty-eight percent of participants were married, 80% were at least high school educated and 31% suffered from other chronic diseases. The mean duration of dermatological disease was 6.1 years (standard deviation = 6.5). There were 192 patients with inflammatory dermatosis and 33 patients with isolated skin lesions. From the results of independent t test/Pearson chi-square test/Fisher’s exact test, all the demographic characteristics of the two groups were not significantly different at the significant level 0.05. Further information of the patients are summarized in Table 3. Out of these, 4 patients had more than 25% of items of Skindex-29 missing, and 9 patients had more than 25% of items of Skindex-16 missing. Hence, the analysis of Skindex-29 and Skindex-16 was carried out on 221 (98%) and 216 (96%) patients respectively.

**Table 3:** **Demographic characteristics of the sample**

**Characteristics**	**Patients with inflammatory dermatoses (** ***N*** **= 192)**	**Patients with isolated lesions (** ***N*** **= 33)**	**Total**
Age, year, mean(SD)^a^	32.9(12.6)	29.7(8.6)	32.5(12.2)
Gender, male (%)^b^	92(47.9)	14(42.2)	106(47.1)
Ethnicity (%)^c^			
Han	174	30	204(90.7)
Others	18	3	21(9.3)
Marital status (%)^c^			
Married or living with partner	95	13	108(48.0)
Single	93	18	111(49.3)
Separated or divorced	4	1	5(2.2)
Widowed	0	1	1(0.4)
Education level (%)^c^			
Grade 9 or less	37	4	41(18.3)
High school	59	12	71(31.6)
College	81	17	98(43.6)
Higher than college	15	0	15(6.7)
Smoke, yes (%)^b^	34(17.7)	3(9.1)	37(16.4)
Drink wine, yes (%)^b^	56(29.2)	9(27.3)	65(28.9)
Exercise, yes (%)^b^	88(45.8)	16(48.5)	104(46.2)
Other chronic disease, yes (%)^b^	60(31.3)	9(27.3)	69(30.7)
BMI, mean(SD)^a^	22.1(3.7)	21.6(3.4)	22.0(3.6)
Duration, year, mean(SD)^a^	6.3(6.3)	5.9(4.8)	6.1(6.5)

SD, Standard Deviation; BMI, Body Mass Index.

^a^Independent t test.

^b^Pearson chi-square test.

^c^Fisher 's exact test.

#### Response distribution and reliability

The means, standard deviations, percentiles, corrected item-total correlations, and Cronbach’s alpha coefficients are presented in Table 4 for Skindex-29 and Skindex-16 and their scales. All scales of Skindex-29 had small floor and ceiling effects (≤5%), while emotions and functioning scales of Skindex-16 had mild floor and ceiling effects (>10%). The Cronbach’s alpha coefficients of Skindex-29 and Skindex-16 and their scales were high (>0.80), and the item-total correlation ranged from 0.5 to 0.88, which signified very good internal-consistency reliability of the two instruments.

**Table 4:** **Descriptive information and Cronbach’s alpha of Skindex-29 and Skindex-16**

	**No. of items (score range)**	**Mean**	**SD**	**Percentile**			**% floor**	**% ceiling**	**Corrected item-total correlation**	**Cronbach’s α**
				**25**	**50**	**75**				
Chinese version of Skindex-29										
Emotions	10(0–100)	49.0	25.0	30.0	50.0	67.5	3.2	0.9	0.63-0.82	0.94
Symptoms	7(0–100)	37.2	21.1	17.9	35.7	53.6	3.2	0.0	0.50-0.67	0.85
Functioning	12(0–100)	36.8	24.6	14.6	35.4	55.2	5.0	0.0	0.56-0.84	0.95
Total	29(0–100)	41.1	22.1	23.7	40.5	55.2	1.4	0.0	0.50-0.83	0.97
Chinese version of Skindex-16										
Emotions	7(0–100)	54.4	31.1	28.6	54.8	83.3	2.8	11.1	0.73-0.88	0.94
Symptoms	4(0–100)	36.2	28.2	12.5	29.2	50.0	8.3	3.7	0.62-0.78	0.86
Functioning	5(0–100)	36.1	30.1	6.7	30.0	60.0	15.3	6.5	0.83-0.88	0.94
Total	16(0–100)	44.3	27.2	21.4	42.7	64.6	2.3	2.3	0.60-0.85	0.96

SD, Standard Deviation.

#### Known-group validity

Patients with inflammatory dermatosis had significantly higher scale and total scores of Skindex-29 and Skindex-16 compared with patients with isolated lesions (Table 5).

**Table 5:** **Variations in total and scale scores of Skindex-29 and Skindex-16 in patients with inflammatory dermatosis and patients with isolated skin lesions**

	**Patients with inflammatory dermatoses mean ± SD**	**Patients with isolated lesions mean ± SD**	***P*** **value**
Skindex-29			
*N*	191	30	
Emotions	52.13 ± 23.98	29.17 ± 22.09	<0.001
Symptoms	39.93 ± 20.46	19.70 ± 16.74	<0.001
Functioning	38.66 ± 24.11	25.13 ± 24.91	0.005
Total	43.61 ± 21.43	25.20 ± 20.07	<0.001
Skindex-16			
*N*	190	26	
Emotions	57.32 ± 30.42	33.04 ± 27.80	<0.001
Symptoms	38.56 ± 27.49	16.19 ± 22.74	<0.001
Functioning	37.65 ± 30.84	24.36 ± 29.09	0.039
Total	46.48 ± 26.55	26.08 ± 23.46	<0.001

SD, Standard Deviation.

**Figure 1:**
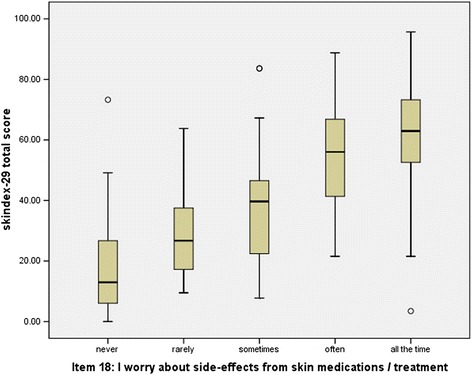
**Box plot of Skindex-29 total scorein patients with various grades of bothered by the side-effects of treatment.**

For the item 18 of Skindex-29 assessing side-effects of treatment, there were 30 patients never worried, 35 patients rarely worried, 66 patients sometimes worried, 55 patients often worried and 35 patients worried all the time. The total score of Skindex-29 showed significantly different among the five groups (Kruskal-Wallis H test Chi-square = 93.39, *P* < 0.001) and increased with the increasing concern of side-effects (Figure 1).

#### Convergent validity

It was demonstrated that moderate to good correlations between Skindex-29 and DLQI, Skindex-16 and DLQI (Skindex-29, *r* from 0.43 to 0.84; Skindex-16, *r* from 0.39 to 0.83) (Table 6).

**Table 6:** **Spearman’s rank correlation coefficients between Skindex-29 and Dermatology Life Quality Index**

	**DLQI**	**Symptoms and feelings**	**Daily activities**	**Leisure**	**Work and school**	**Personal relationship**	**Treatment**
Skindex-29							
Emotions	0.75	0.63	0.62	0.66	0.46	0.43	0.51
Symptoms	0.64	0.57	0.58	0.54	0.62	0.72	0.62
Functioning	0.84	0.64	0.70	0.72	0.59	0.67	0.62
Total score	0.83	0.67	0.70	0.71	0.51	0.61	0.55
Skindex-16							
Emotions	0.70	0.64	0.58	0.58	0.52	0.51	0.48
Symptoms	0.60	0.58	0.55	0.47	0.44	0.41	0.39
Functioning	0.83	0.63	0.70	0.75	0.64	0.71	0.57
Total score	0.81	0.70	0.69	0.69	0.61	0.62	0.56

DLQI, Dermatology Life Quality Index.

*P* < 0.001 for all.

#### Factor structure validity

Exploratory factor analysis using eigenvalues greater than 1 as the criterion resulted in 4 factors for Skindex-29 and 3 factors for Skindex-16, which accounted for 68.66% and 77.78% of the total variances, respectively. With oblique rotation method, the factor loadings of Skindex-29 and Skindex-16 are presented in Tables 7 and 8. For Skindex-29, the first, second and fourth factors matched almost the three original scales: Functioning, Emotion and Symptoms. The third factor, however, seemed as the combination of items originally belonging to the Function and Symptoms. For Skindex-16, the extracted three factors reflected almost perfectly the original scales of functioning, emotion and symptoms. And the factor loadings ranged from 0.799 to 0.969 for factor 1 (functioning), 0.459 to 0.998 for factor 2 (emotion) and 0.637 to 0.882 for factor 3 (symptoms) respectively.

**Table 7:** **Exploratory factor loadings of Skindex-29 items**

**Items**	**Factor 1 (eigenvalue = 15.613)**	**Factor 2 (eigenvalue = 1.828)**	**Factor 3 (eigenvalue = 1.441)**	**Factor 4 (eigenvalue = 1.031)**
*Functioning*				
2. My skin condition affects how well I sleep	0.056	0.014	**0.779**	−0.079
4. My skin condition makes it hard to work or do hobbies	0.327	0.183	**0.583**	−0.106
5. My skin condition affects my social life	**0.624**	0.168	0.117	0.077
8. I tend to stay at home because of my skin condition	**0.556**	0.153	0.173	0.055
11. My skin condition affects how close I can be with those I love	**0.776**	−0.189	0.260	0.032
14. I tend to do things by myself because of my skin condition	**0.703**	0.052	0.051	0.115
17. My skin condition makes showing affection difficult	**0.785**	−0.091	0.094	0.148
20. My skin condition affects my interactions with others	**0.766**	0.105	−0.012	0.124
22. My skin condition is a problem for the people I love	**0.522**	0.082	0.322	0.006
25. My skin condition affects my desire to be with people	**0.667**	0.205	0.045	−0.030
29. My skin condition interferes with my sex life	**0.664**	−0.383	0.517	0.009
30. My skin condition makes me tired	0.281	**0.479**	0.298	−0.052
*Emotion*				
3. I worry that my skin condition may be serious	0.129	**0.445**	0.384	0.030
6. My skin condition makes me feel depressed	0.266	**0.636**	0.145	−0.141
9. I worry about getting scars from my skin condition	0.251	**0.710**	−0.464	0.146
12. I am ashamed of my skin condition	**0.747**	0.299	−0.148	−0.016
13. I worry that my skin condition may get worse	0.179	**0.626**	0.068	−0.022
15. I am angry about my skin condition	0.325	**0.540**	−0.050	0.080
21. I am embarrassed by my skin condition	**0.649**	0.387	−0.148	−0.010
23. I am frustrated by my skin condition	**0.423**	**0.515**	0.050	−0.094
26. I am humiliated by my skin condition	**0.808**	0.213	−0.228	0.033
28. I am annoyed by my skin condition	0.271	0.680	0.129	−0.233
*Symptoms*				
1. My skin hurts	0.010	0.077	0.237	**0.573**
7. My skin condition burns or stings	0.045	−0.079	**0.517**	**0.475**
10. My skin itches	−0.058	0.112	**0.736**	0.030
16. Water bothers my skin condition (bathing, washing hands)	0.242	−0.063	−0.168	**0.792**
19. My skin is irritated	−0.318	**0.729**	0.201	0.315
24. My skin is sensitive	−0.325	**0.633**	0.430	0.113
27. My skin condition bleeds	0.158	0.154	0.026	**0.547**

Bold values indicate the largest factor loadings for each item.

**Table 8:** **Exploratory factor loadings of Skindex-16 items**

**Items**	**Factor 1 (eigenvalue = 9.675)**	**Factor 2 (eigenvalue = 1.454)**	**Factor 3 (eigenvalue = 1.316)**
*Functioning*			
12. The effects of your skin condition on your interactions with others (For example: interactions with family, friends, close relationships, etc.)	**0.969**	−0.089	0.023
13. The effects of your skin condition on your desire to be with people	**0.854**	0.012	0.068
14. Your skin condition making it hard to show affection	**0.964**	−0.129	0.048
15. The effects of your skin condition on your daily activities	**0.809**	−0.031	0.141
16. Your skin condition making it hard to work or do what you enjoy	**0.799**	0.065	0.058
*Emotion*			
5. The persistence/reoccurrence of your skin condition	−0.194	**0.836**	0.257
6. Worry about your skin condition (For example: that it will spread, get worse, scar, be unpredictable, etc.)	−0.210	**0.998**	0.030
7. The appearance of your skin condition	0.083	**0.776**	0.010
8. Frustration about your skin condition	**0.500**	**0.578**	−0.163
9. Embarrassment about your skin condition	**0.613**	0.459	−0.131
10. Being annoyed about your skin condition	0.267	**0.742**	−0.036
11. Feeling depressed about your skin condition	0.302	**0.689**	−0.005
*Symptoms*			
1. Your skin condition itching	−0.009	0.156	**0.762**
2. Your skin condition burning or stinging	0.092	−0.064	**0.882**
3. Your skin condition hurting	0.245	−0.128	**0.769**
4. Your skin condition being irritated	−0.122	0.333	**0.637**

Bold values indicate the largest factor loadings for each item.

**Figure 2:**
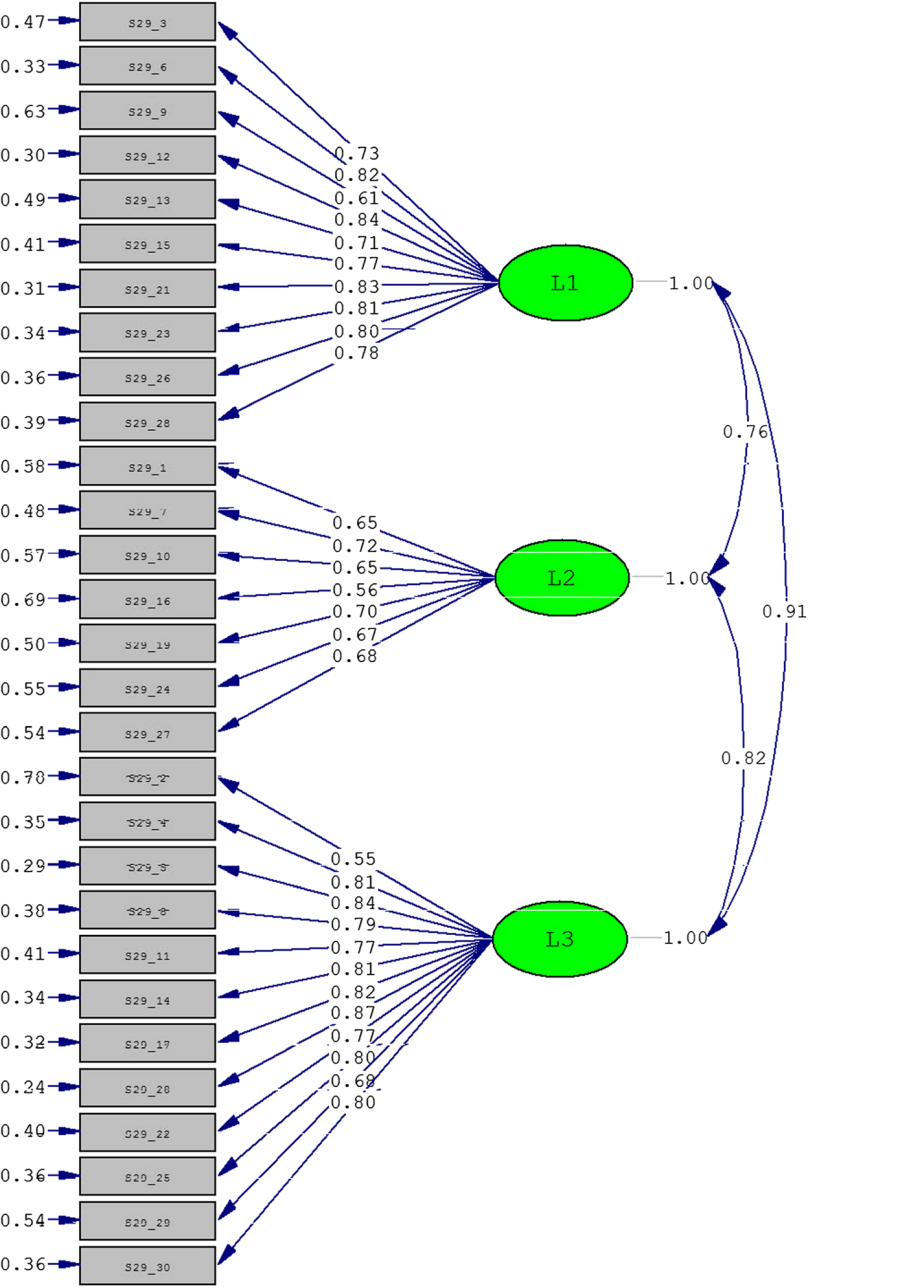
**The three-factor model for Skindex-29 obtained from confirmatory factor analysis.**

**Figure 3:**
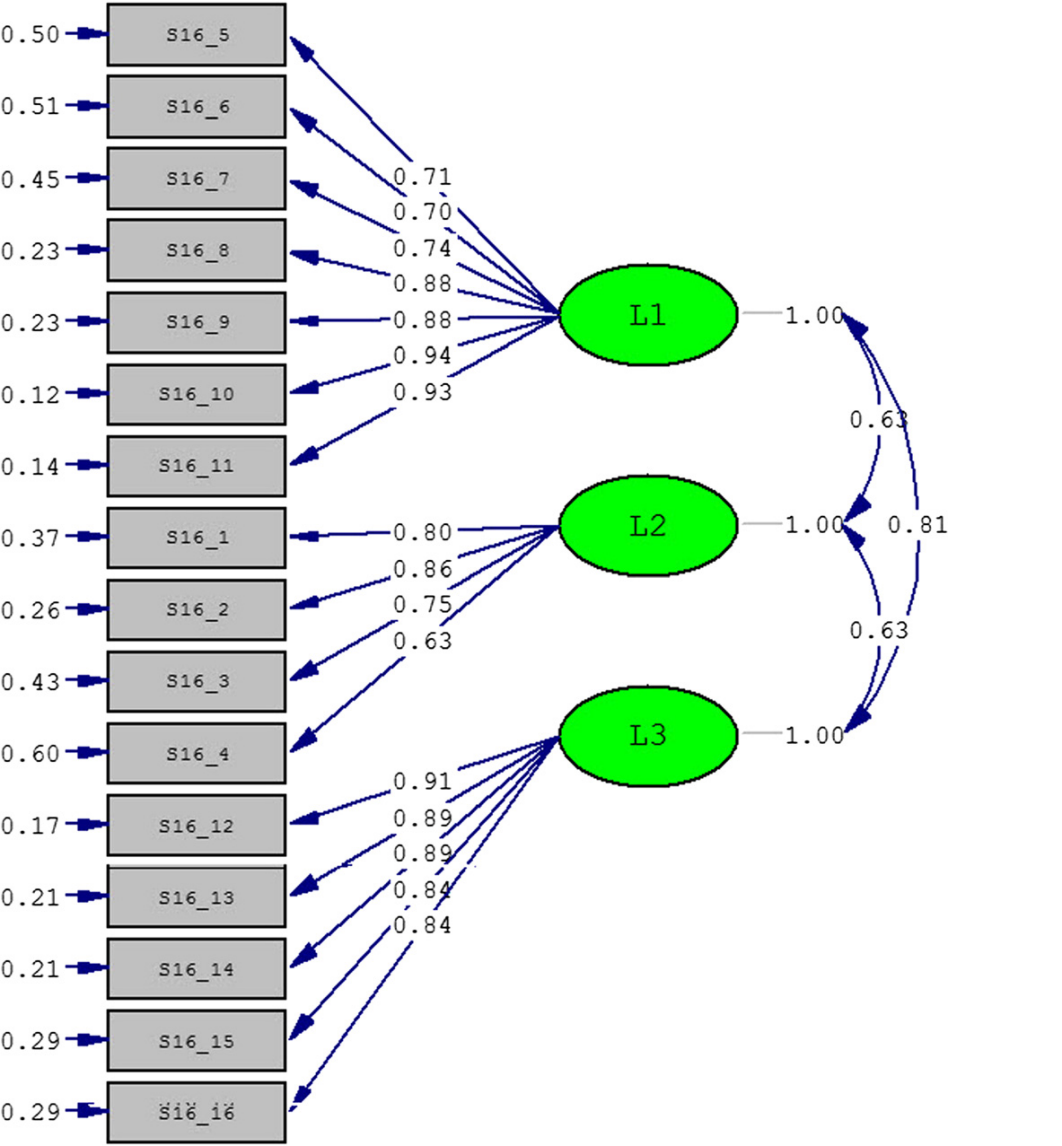
**The three-factor model for Skindex-16 obtained from confirmatory factor analysis.**

Confirmatory factor analysis was also performed to evaluate the factor structure validity of Skindex-29 and Skindex-16 according to their original structure. The goodness-of-fit indices are shown in Table 9, and the structure graphs are shown in Figures 2 and 3 respectively for Skindex-29 and Skindex-16. The two instruments got similar results. The indices GFI, AGFI and RMSEA were not as good as possible, but the NNFI and CFI were large enough to meet the cut-off criterion of goodness-of-fit indices (lager than 0.90). Meanwhile, the SRMR, which was regarded as the most important index, was also satisfying (less than 0.08) [16].

**Table 9:** **Goodness-of-fit indices of three-factor structure model for Skindex-29 and Skindex-16**

	**GFI**	**AGFI**	**NNFI**	**CFI**	**SRMR**	**RMSEA (90% Confidence interval)**
Skindex-29	0.70	0.66	0.96	0.96	0.06	0.11 (0.10, 0.11)
Skindex-16	0.74	0.65	0.94	0.95	0.07	0.15(0.14, 0.17)

## Discussion

In this study, we translated Skindex-29 and Skindex-16 into Chinese according to accepted steps in the generation of conceptually and linguistically equivalent translations of quality of life measures. Our evaluation of the psychometric properties of the resulting Chinese versions of Skindex-29 and Skindex-16 suggested that they are reliable and valid measures of HrQoL in Chinese patients with dermatological disease.

Our translation steps were similar with those of other language versions of Skindex-29 and Skindex-16 [17-20]. However, the evaluation of the backward translation and the definition of the final version were completed by the original author instead of a multidisciplinary committee suggested by the guidelines for cross-cultural adaption of health-related quality of life measures [10], which might lead to potential bias. During the translation process, some words relating emotions, like “embarrassed”, “ashamed”, “frustrated” became the most difficult and problematic issue for expression in Chinese. The Chinese translation of these words was modified until a satisfactory version was created. Similar problem was encountered, for example, in the development of Turkish version of Skindex-29 [18].

With respect to the reliability, the Cronbach’s alpha coefficients for the global and three scales were all greater than 0.80, which indicated satisfactory internal consistency of the Chinese version of Skindex-29 and Skindex-16. And the results were similar to those of other language versions of Skindex-29 and Skindex-16 [17-20]. High item-total correlations further confirmed the good homogeneity of the questionnaires. Mild floor and ceiling effects were noted for the scales of functioning and emotions of Skindex-16, while no floor or ceiling effect was observed for Skindex-29. It might suggest that Skindex-16 provide limited information on bothering by dermatological disease for those with low level of functioning and high level of emotions.

Our results showed that the Chinese version of Skindex-29 and Skindex-16 had good capacity to discriminate patients with inflammatory dermatosis and patients with isolated lesions, which was consistent with the original version and other language versions [17-20]. Furthermore, we found a difference in the total score of Skindex-29 among patients with different levels of being bothered by the side-effects of treatment which was measured by the item 18 of Skindex-29. The more bothered by the side-effects of treatment, the worse quality of life patients had.

The pattern of correlation between Skindex-29 and Skindex-16 scale scores and DLQI subscale scores provides available evidence of convergent validity. For all three scales, they exhibited moderate to excellent correlations with the six subscales of DLQI. The reasons of choosing DLQI as the measures that Skindex should theoretically be related to are that the DLQI is the most commonly used HrQoL instruments in dermatology and its Chinese version has been proved to be reliable and valid [12,21].

The exploratory factor analysis yielded four factors for Skindex-29 and three factors for Skindex-16. The result of Skindex-29 was consistent with that of the Polish version [22], but in contrast to the three-factor structure extracted by the original version and Italian version [4,23]. The differences between present results and the results in the US and Italy samples might be partly due to differences in sample characteristics. The results of Skindex-16 were in accordance with the original version and provided further confirmation of the three factors of the instrument.

There has not been confirmatory factor analysis of Skindex, and our results indicated that the three-factor structure of Skindex-29 and Skindex-16 was acceptable. The reason of low values of GFI and AGFI and high value of RMSEA might be that they could be impacted by the sample size which in this study was relatively small [16].

There were some limitations in this study. The study sample was from the outpatients of the department of dermatology and the time consuming of completing the questionnaire might lead to selection bias. To decrease burden of the study on patients, test-retest reliability was not evaluated. And responsiveness, i.e. the ability of the instrument to detect small but meaningful changes over time was not evaluated either because it was hard to investigate clinical significant changes for many dermatologic conditions enrolled in this study within a reasonable time frame.

Additional studies would include further validation with patients from multi-center clinics, evaluation of test-retest reproducibility and responsiveness of the instruments. And international cooperative research could be expected to use the Skindex to measure HrQoL in patients with dermatological disease. In addition, more information on patients from different socio-cultural areas and environments would be desirable. In particular, using the original English version of scale could have introduced response bias because of cultural differences between the US and China. How the Skindex might exhibit Differential Item Functioning (DIF) among patients with different cultural background needs further research.

## Conclusion

The Chinese versions of Skindex-29 and Skindex-16 have been developed and demonstrated to be reliable and valid instruments for use as HrQoL instruments in Chinese patients with dermatological disease. The Skindex is expected to be adopted into clinical practice and clinical trials to allow physicians to increase the attention paid to HRQoL of patients not only the symptoms of dermatological disease.

## Abbreviations

HrQoL: Health-related quality of life
DLQI: Dermatology life quality index
EFA: Exploratory factor analysis
CFA: Confirmatory factor analysis
GFI: Goodness of fit index
AGFI: Adjusted goodness of fitindex
NFI: Normed fit index
CFI: Comparative fit index
SRMR: Standardized root mean square residual
RMSEA: Rootmean square error of approximation
